# Healthier Fundraising in U. S. Elementary Schools: Associations between Policies at the State, District, and School Levels

**DOI:** 10.1371/journal.pone.0049890

**Published:** 2012-11-14

**Authors:** Lindsey Turner, Jamie F. Chriqui, Frank J. Chaloupka

**Affiliations:** 1 Health Policy Center, Institute for Health Research and Policy, University of Illinois at Chicago, Chicago, Illinois, United States of America; 2 Department of Political Science, University of Illinois at Chicago, Chicago, Illinois, United States of America; 3 Department of Economics, University of Illinois at Chicago, Chicago, Illinois, United States of America; Indiana University, United States of America

## Abstract

**Objectives:**

We examined whether state laws and district policies pertaining to nutritional restrictions on school fundraisers were associated with school policies as reported by administrators in a nationally-representative sample of United States public elementary schools.

**Methods:**

We gathered data on school-level fundraising policies via a mail-back survey during the 2009–10 and 2010–11 school years. Surveys were received from 1,278 public elementary schools (response rate = 60.9%). Data were also gathered on corresponding school district policies and state laws. After removing cases with missing data, the sample size for analysis was 1,215 schools.

**Results:**

After controlling for school characteristics, school policies were consistently associated with state laws and district policies, both those pertaining to fundraising generally, as well as specific restrictions on the sale of candy and soda in fundraisers (all Odds Ratios >2.0 and *P*s<.05). However, even where district policies and state laws required fundraising restrictions, school policies were not uniformly present; school policies were also in place at only 55.8% of these schools, but were more common at schools in the West (77.1%) and at majority-Latino schools (71.4%), indicating uneven school-level implementation of district policy and state law.

**Conclusions:**

District policies and state laws were associated with a higher prevalence of elementary school-level fundraising policies, but many schools that were subject to district policies and state laws did not have school-level restrictions in place, suggesting the need for further attention to factors hindering policy implementation in schools.

## Introduction

In recent years, the school food environment has been a focal point in efforts to reverse the childhood obesity epidemic [1]–[4]. With recent estimates showing that one-third of children ages 6–11 years old in the United States (U.S.) were overweight or obese during 2009–10 [5], continued attention to these effort is needed. Foods and beverages sold in schools are generally broken into two categories: 1) school meals; and 2) competitive foods, which include all foods and beverages sold or offered to students outside of the meals programs. Competitive products typically include items sold in vending machines, school stores and snack bars, or à la carte in the cafeteria, through in-school fundraisers, and offered to students during classroom parties and as rewards in the classroom [6].

Competitive foods and beverages are widely available in schools [7], [8]. Nationwide data from the 2009–10 school year indicate that 65% of U.S. public elementary-school students could purchase foods or beverages from competitive venues (vending machines, school stores/snack bars, and or à la carte lines) [9]. The third School Nutrition and Dietary Assessment Study (SNDA-III), conducted during the 2004–05 school year, indicated that 73% of public elementary schools offered at least one source of competitive foods and beverages, including the traditional sales venues (vending machines, school stores/snack bars, and à la carte lines) as well as fundraisers, parties, and given as rewards in the classroom; furthermore, 29% of elementary school students consumed competitive items during the average school day [10]. Although many studies have examined the availability of competitive foods and beverages in tradition sales venues [10]–[18], less work has examined the prevalence and characteristics of school fundraising activities, or the extent to which school fundraising practices are associated with policy restrictions on items sold through fundraisers.

Nationwide data suggest that school fundraisers are common in the U.S., and, therefore, are an important component of any strategy to change the competitive food environment in schools. SNDA-III results from 2004–05 indicated that classroom parties, bake sales and other fundraisers were a common source of competitive foods for elementary school students [10], with 37% of public elementary schools holding some sort of fundraiser [7]. Higher estimates were obtained by the Centers for Disease Control and Prevention's nationally-representative School Health Policies and Programs Study 2006, which found that at 76% of elementary schools, an organization such as the parent-teacher-association sold foods or beverages at school or in the community to raise money [12]. Specifically in elementary schools, the items sold were chocolate candies (50% of schools), baked goods (50% of schools) and sugar-sweetened beverages (21% of schools) [12].

Research is accumulating to demonstrate that policies regarding competitive food and beverages—including those sold as well as those offered in the classroom and in other ways on campus—are significantly associated with children's diets and weight status, and a recent comprehensive research review documents the importance of such policies in schools [19]. Although, the evidence specifically relating to fundraisers is fairly limited thus far, research suggests that school fundraisers may be associated with student weight outcomes. Kubik and colleagues [20] found that in Minnesota middle schools, school practices—including fundraising—were associated with student body mass index (BMI). Higher scores on a school food practices scale that included classroom and school-wide fundraising activities (as well as allowing foods and beverages in the classroom, snacks and beverages in hallways, and food-based rewards) were associated with higher student BMI scores [20]. However, the specific association between fundraising activities and student BMI was not examined.

With regard to strategies for improving school fundraising practices, some work in secondary schools has established a link between fundraising policies and school practices. In a study of 45 middle schools and 71 high schools in the Midwest during 2006, 37% of schools used chocolate, candy, and high-fat baked goods for school-wide fundraising activities, and 50% of schools had policies addressing the nutritional quality of foods and beverages used in fundraising [21]. Healthful fundraising policies were associated with school practices, and there was a greater concordance between school fundraising policies and practices in middle schools than in high schools [21]. However, while it is clear that fundraising activities are a source of high-calorie foods and beverages in elementary schools [10], thus far little attention has been paid to the relationship between policy efforts to change school food-related fundraising and implementation at the elementary-school level. State-specific research has shown that policy strategies can impact school practices. For example, the Arkansas Act 1220 of 2003—one of the first and most comprehensive state laws to combat childhood obesity—created a statewide health advisory committee to develop specific recommendations for nutrition in schools, and also required school districts to establish committees to develop locally-relevant policies for schools. Subsequently, the prevalence of school policies prohibiting the use of food in fundraising by student groups increased significantly, from 54% to 70% of elementary schools [22].

In recent years, increasing attention has been directed to the potential for state laws and school district policies to play a role in promoting healthy school environments. In the U.S., the *Child Nutrition and WIC Reauthorization Act of 2004* [23] mandated that school districts participating in federal child nutrition programs (i.e., school breakfast and lunch programs, in which most public school districts participate) must adopt and implement a wellness policy by the first day of the 2006–07 school year; these policies were to include nutrition guidelines for all foods, including competitive foods and beverages. Each local school district was responsible for establishing the guidelines, allowing for much variability in policy focus, strength and implementation. Since the wellness policy mandate went into effect, policies to address competitive foods and beverages have increased in prevalence, but as of the 2008–09 school year, only 36% of public elementary school students nationwide were enrolled in a district that restricted fundraisers during the school day [17]. Further refinements in district policies are expected, following the *Healthy, Hunger-Free Kids Act of 2010* [24], which continues to require district wellness policies. This legislation also directed the United States Department of Agriculture (USDA) to develop nationwide regulations for competitive foods and beverages (although the federal rule will not preempt stronger state and/or local laws/policies). Currently, the only USDA regulation regarding competitive foods is a prohibit on the sale of foods of minimal nutritional value (FMNVs)—a category that includes carbonated soft drinks and sodas, as well as certain candies— in the cafeteria during meals, but these items may be sold elsewhere at school even during lunch [6].

In the meantime, however, there is scant data on whether—or how—state law and/or district policy efforts nationwide are associated with school-level policies and practices. The current study uses cross-sectional data from a nationally-representative sample of U.S. public elementary schools during the 2009–10 and 2010–11 school years, along with corresponding district policies and state laws, to examine the associations between state laws, district policies, and school-level fundraising restrictions.

## Methods

Data on school practices were gathered via mail-back survey. Data on corresponding district policies and state laws were gathered via commercially available public-use sources as described below. This study was reviewed and approved by the Institutional Review Board at the University of Illinois at Chicago. A waiver of documentation of informed consent was granted, as consent was implied by return of the survey.

### School-Level Data

#### Sampling and Weighting

For each year, a different sample of schools was used, and each of the samples were developed at the Institute for Survey Research at the University of Michigan and were designed to be nationally-representative of U.S. public elementary schools (containing a 3^rd^ grade) from the contiguous states (excluding Alaska and Hawaii). School weights were developed and adjusted for non-response bias.

#### Procedure

Surveys were mailed to school principals in January of each school year, with subsequent follow-up by mail, e-mail and telephone until recruitment ended in June of each year. The instructions requested that the survey be completed by the principal or other staff with knowledge of school practices pertaining to student health. A $100 incentive was offered to the respondent or school for returning the survey. The response rate—calculated using the American Association for Public Opinion Research Method Two [25]—was 60.9% (n = 1278 schools); this was slightly higher in 2009–10 (64.5%; n = 680 schools) than in 2010–11 (57.4%; n = 598 schools). Surveys were processed and double-entered for quality assurance.

#### Measures

Information on school fundraising restrictions was gathered with three survey items. The first item asked respondents to indicate “Does your school have any school-wide policies regarding the nutritional quality of items sold for PTA fundraisers or other fundraisers?” with responses of “Yes” (coded = 1), “No” (coded = 0) or “N/A, no fundraising.” Two additional items asked which of the following types of restrictions were present: 1) No foods of minimal nutritional value (FMNVs; carbonated soft drinks and certain candies) and 2) No soda/soft drinks. These items were coded 1 = “Yes” versus 0 = “No” or “No restrictions.” For the current analyses, the 5.1% of schools (n = 65) that did not allow any fundraising were re-coded as 1 = yes for each of the fundraising items (i.e., complete ban).

#### Contextual Factors

School-level demographic and socioeconomic data were obtained from public use data files from the National Center for Education Statistics (Common Core of Data 2009–10). These variables were used as covariates in regression analyses. Data were obtained on all available school characteristics, coded as follows: school size (total number of students in the school), coded in tertiles as small (<451 students), medium (451–621 students), and large (>621 students; referent); the percentage of students eligible for free or reduced-price lunch (as a proxy for socioeconomic status; SES), coded in thirds as lower SES (>66%), medium SES (≤66% to >33%), and higher SES (≤33%; referent); U.S. census region, coded as West, Northeast, Midwest and South (referent); and locale, coded as rural, town, suburban, and urban (referent). For racial/ethnic composition each school was coded as one of four exhaustive and mutually-exclusive categories: majority (≥66%) White students (referent); majority (≥50%) Latino students; majority (≥50%) Black students; and diverse (no majority).

### District-Level Policy Data Collection

Formal policy documents (i.e., wellness policies, associated rules/regulations, and other policies embedded by reference into the wellness policy/rules) were collected from the corresponding school district for each elementary school in the sample. Policies were gathered by trained research assistants using an established protocol via internet searches, with telephone calls and/or mailings to find policies that were unavailable online and to verify complete policy collection for policies that were available online. All district policies were reviewed and double-coded and analyzed by two trained researchers using an adaptation of a coding scheme developed by Schwartz and colleagues [26] and presented by Chriqui and colleagues [27], [28]. After double-coding was complete, a consensus review was conducted to discuss any discrepancies (<5% for these topics).

Policy provisions pertaining to fundraisers were coded as: 0 = no policy, if there were no fundraising food/beverage standards; 1 = weak policy, if restrictions on items sold for fundraising were vague, suggested but not required, only applied to a limited set of products (e.g., FMNVs), or applied for less than the whole school day; 2 = strong policy, if specific fundraising nutrition standards were required; and 3 = complete ban, if all competitive foods were banned. In addition, policy codes for two specific nutrition standards were used in the current analyses, candy and regular soda, each coded for whether the policy applied specifically to items sold as fundraisers. The candy item was coded as: 0 = no policy; 1 = weak policy, if the item was restricted but not prohibited; 2 = strong policy that prohibited candy in fundraisers; and 3) complete ban on competitive foods or fundraisers. The soda item was coded using a five-level variable: 0 = no policy; 1 = weak policy, if the item was restricted but not prohibited; 2 = regular soda prohibited but not all sugar-sweetened beverages; 3 = all sugar-sweetened beverages prohibited; and 4 = competitive beverage or fundraising ban. District policy variables were collapsed such that 1 = any strong/required policy (original codes of 2, 3, or 4, as appropriate) versus 0 = no policy or a weak policy (original codes of 0 and 1).

### State Law Data Collection

State laws, effective as of the beginning of September of each school year were compiled through natural language and Boolean keyword searches of the full-text, tables of contents, and indices of codified state statutory and administrative (regulatory) laws commercially available from subscription-based legal research providers, Westlaw and Lexis-Nexis. Codified state statutory laws include legislation enacted by the state legislatures while codified administrative laws include all rules/regulations promulgated by state Boards of Education. For purposes of this study, all those legislative laws and rules/regulations that were formally codified were included for this analysis. Any “informal” policies adopted by state Boards of Education, for example, that were not codified into rule/regulation were excluded from this study. The vast majority of all such rules/regulations are codified into law and are included herein. The codified state laws (including regulations) were validated against publicly available secondary sources including the National Conference of State Legislatures, the National Association of State Boards of Education, and the National Cancer Institute's Classification of Laws about School Students [29]–[31]. State laws were coded using the same methods and coding rubric as described above for district policies.

### Data Analysis

The initial sample included 1278 schools. At 31 schools, the survey item on school-level fundraising restrictions was skipped, and at 28 schools, district policy data were not available (schools within 26 unique districts). At four schools, data were unavailable on free and reduced lunch eligibility (a covariate), thus the final sample size for analyses reported here was reduced to 1215 schools. The samples were selected as separate cross-sectional samples for each year, but a small amount of overlap occurred between the two years; nine schools responded in both years, along with 1198 unique schools (n = 649 in 2009–10 and n = 548 in 2010–11). Analyses were conducted with the data treated as a stacked cross-sectional dataset, controlling for year.

Analyses were conducted using complex survey commands in STATA/SE 12.0 [32] and accounted for the clustering of schools within districts and states. Data were weighted to provide inference to all public elementary schools in the U.S. First, the sample characteristics were tabulated (Table 1). Then the prevalence of school policy status was tabulated; based on cross-tabulation of the collapsed (binary) district policy variables and the binary state law variables, each school was classified into one of four mutually exclusive and exhaustive categories for each fundraising policy category of interest (i.e., overall fundraising restrictions, no sodas in fundraisers, no candy in fundraisers): 1) no district policy and no state law; 2) district policy only; 3) state law only; and 4) both state law and district policy (Table 2). Then, the relationships between state law and school policies and between district and school policies were examined with a series of multivariate logistic regressions that included a set of three variables to account for district policy and state law (Table 3). For regression analyses, a set of three dummy codes compared each of the three latter categories against ‘no policy’ (referent). Outcomes were coded so that 1 = the presence of a school-level nutritional restriction on fundraisers and 0 = no restriction. Each school variable was matched to the relevant policy dimension (i.e., policies pertaining to candy and soda were used to predict school-level restrictions on FMNVs). Table 3 also presents predicted margins, which equal the adjusted prevalence of schools that placed restrictions on fundraisers within each policy category (i.e., no policy, district-only, state-only, and both district and state), controlling for covariates. Standard errors were adjusted to account for the sampling variability of the covariates in the model.

**Table 1:** Characteristics of the school sample, 2009–11 school years.

Characteristic	% Schools
***Fundraising Outcomes***	
Any fundraising restrictions	39.2
No FMNVs	25.1
No soda	21.7
***School Demographics***	
Region	
South	35.1
Northeast	16.8
Midwest	25.1
West	23.1
Locale	
City	31.9
Suburb	30.6
Town	11.0
Rural	26.5
Race/ethnicity of students	
Majority (≥66%) White	47.4
Majority (≥50%) Black	11.0
Majority (≥50%) Latino students	17.4
Diverse	24.2
Student eligibility for free or reduced- price lunch	
Lowest (≤33% eligible)	25.7
Medium (>33% to ≤66% eligible)	37.2
Highest (>66% eligible)	37.1
School size	
Large (>621 students)	20.3
Medium (>450 to 621 students)	30.4
Small (≤450 students)	49.2

Percentages sum to 100 within category, but due to rounding may not sum to exactly 100.

Data are weighted to the school level, n = 1215 schools.

**Table 2:** Percentages of schools in districts and/or states with policies/laws restricting school fundraising activities, 2009–11 school years.

Policy Category	% Schools
Overall fundraising restrictions	
None (ref)	59.9
District only	7.9
State only	5.4
State and district	26.8
Fundraising prohibition on candy	
None (ref)	79.7
District only	5.3
State only	1.7
State and district	13.3
Fundraising prohibition on soda	
None (ref)	52.0
District only	10.9
State only	6.7
State and district	30.4

Percentages sum to 100 within category, but due to rounding may not sum to exactly 100.

Data are weighted to the school level, n = 1215 schools.

**Table 3:** Summary of results of multivariate logistic regression models to predict school-level fundraising practices.

	Outcomes: School-Level Fundraising Restrictions					
	Any restrictions		No FMNVs		No soda	
State/District Predictors	Adjusted^a^ Odds Ratio(95% CI)	Adjusted Prevalence^b^M (SE)	Adjusted^a^ Odds Ratio(95% CI)	Adjusted Prevalence^b^M (SE)	Adjusted^a^ Odds Ratio(95% CI)	Adjusted Prevalence^b^M (SE)
Overall fundraising restrictions						
None	Ref	0.31 (0.02)	Ref	0.19 (0.02)	Ref	0.16 (0.02)
District only	2.02 (1.08–3.77)*	0.47 (0.07)	1.32 (0.74–2.35)	0.24 (0.05)	1.50 (0.84–2.70)	0.22 (0.05)
State only	1.16 (0.63–2.15)	0.35 (0.06)	1.53 (0.76–3.07)	0.27 (0.06)	1.63 (0.89–3.00)	0.24 (0.05)
State and district	2.78 (1.89–4.10)***	0.55 (0.03)	2.60 (1.74–3.87)***	0.38 (0.03)	2.61 (1.70–3.98)***	0.33 (0.04)
Fundraising prohibition on candy						
None			Ref	0.23 (0.02)		
District only			1.03 (0.50–2.10)	0.23 (0.06)		
State only			1.17 (0.27–5.12)	0.26 (0.14)		
State and district			2.11 (1.34–3.35)***	0.38 (0.05)		
Fundraising prohibition on soda						
None			Ref	0.19 (0.02)	Ref	0.16 (0.02)
District only			1.47 (0.87–2.51)	0.25 (0.04)	1.70 (1.03–2.81)*	0.24 (0.04)
State only			1.40 (0.70–2.80)	0.24 (0.06)	1.73 (0.92–3.26)	0.24 (0.05)
State and district			2.40 (1.61–3.59)***	0.35 (0.03)	2.47 (1.60–3.82)***	0.31 (0.03)

a All models include school-level covariates: school size (ref: large), locale (ref: city) region (ref: south), student race/ethnicity (ref: majority white), percentage of students eligible for free or reduced-price lunch (ref: lowest), and year (ref: 2009–10).

b Adjusted prevalence represents the percentage of schools with a restriction on fundraising, by state/district policy status, adjusted for all covariates noted above.

Outcomes coded 1 = school-level restriction, 0 = no school-level restriction.

Data include the 2009–10 and 2010–11 school years, analyses weighted to the school level, n = 1215 schools.

* *P*<.05.

*** *P*<.001.

Finally, to examine the characteristics of schools where the school-level policy was or was not consistent with district policies and state laws, Table 4 presents the percentage of schools with or without a school-level policy regarding the nutritional quality of items sold in fundraising, among the schools where district policy and state law both required such restrictions. It also presents the percentage of schools with or without a school-level policy among schools in states where both the district policy and state law did not require such restrictions. These breakdowns compare the percentage of schools with a policy within demographic subgroups.

**Table 4:** Percentage of schools with any school-level fundraising policy or no policy, by school demographic characteristics, for schools where both state laws and district-level fundraising policies are present, and those where policies are absent.

	Strong district policy and state law			No district policy and no state law		
	No school policy (n = 136)	School policy (n = 172)	χ^2^	No school policy (n = 541)	School policy (n = 217)	χ^2^
Overall	42.0	58.0		70.3	29.7	
Region						
South	60.9	39.1		69.4	30.6	
Northeast	35.3	64.7		59.1	40.9	
Midwest	42.1	57.9		72.9	27.1	
West	22.9	77.1	<.0001	77.7	22.3	.0523
Locale						
City	38.4	61.6		69.8	30.2	
Suburb	38.3	61.7		70.5	29.5	
Town	63.8	36.2		64.4	35.6	
Rural	47.7	52.3	.1035	72.8	27.2	.5883
Race/ethnicity of students						
Majority (≥66%) White	55.7	44.3		70.3	29.7	
Majority (≥50%) Black	53.0	47.0		62.9	37.1	
Majority (≥50%) Latino students	28.6	71.4		64.9	35.1	
Diverse	43.2	56.8	.0068	76.2	23.8	.3009
Student eligibility for free or reduced-price lunch						
Lowest (≤33% eligible)	51.0	49.0		72.9	27.1	
Medium (>33% to ≤66% eligible)	43.2	56.8		70.6	29.4	
Highest (>66% eligible)	37.0	63.0	.2821	67.3	32.7	.5609
School size						
Small (<451 students)	39.5	60.5		70.2	29.8	
Medium (451 to 621 students)	44.4	55.6		70.2	29.8	
Large (>621 students)	42.4	57.6	.8232	70.5	29.5	.9977

## Results

As reported by survey respondents at 1215 U.S. public elementary schools, during the 2009–11 school years, 39.2% of schools had some sort of school-wide restriction regarding the nutritional quality of items sold for fundraisers (see Table 1). Percentages by year were 41.8% in 2009–10 and 36.6% in 2010–11; as tested in a multivariate logistic regression including covariates, this did not differ significantly across the two years. Bans on FMNVs were reported at 25.1% of schools (25.0% in 2009–10 and 25.2% in 2010–11), and bans on soda and/or soft drinks were reported at 21.7% of schools (20.8% in 2009–10 and 22.5% in 2010–11).

Across the two years, responding schools were located in 834 unique districts, with schools located in 407 unique districts in 2009–10 only, 383 unique districts in 2010–11 only, and 44 districts were represented for both years. Across those 834 districts, 252 (30.2%) had a strong fundraising policy. Responding schools were located in 47 states (all states except Alaska, Hawaii, Wyoming, and the District of Columbia), with most states (n = 44) represented for both years, two states (North Dakota and Rhode Island) represented for 2009–10 only, and one state (Vermont) represented for 2010–11 only. Among the 47 states represented in one or both years, 10 (21.3%) had a strong law. When examined in combination (Table 2), most schools were located in districts and states with no policy at either level; however, approximately one-fourth of schools were subject to policies at both levels.

Across the multivariate logistic regression models—predicting all three types of fundraising restrictions at schools—schools with a combination of a state law and a district policy were more than two times as likely to have nutritional limitations on fundraisers than were schools where there was no corresponding law or policy (Table 3). Additionally, elementary schools located in districts with overall fundraising restrictions and prohibitions on soda sold through fundraisers were also more likely to restrict fundraisers and prohibit soda, respectively.

Further analysis indicated that school-level policies were not necessarily consistent with state laws and district policies (Table 4). Where state law and district policy required fundraising limitations, still only 55.8% of schools reported having a school-level policy. This varied significantly by region and student race/ethnicity, with school-level policies being concordant with a strong district policy and state law among 77.1% of schools in the West versus 39.1% of schools in the South, and for 71.4% of majority-Latino schools compared with 44.3% of majority-White schools and 47.0% of majority-Black schools (*P*s<.01). Information given in response to an open-ended follow-up question regarding the types of school-level fundraising policies in place indicated that many schools follow relevant state laws and/or district policies; however, in some cases, respondents indicated that although their school was subject to a district wellness policy, those fundraising restrictions were not always followed by the school. As also shown in Table 4, school-level policies were reported by 29.7% of schools located in states with no law and districts with no policies, suggesting that some schools are developing policies independently; this did not vary by school demographic characteristic.

## Discussion

Our results show that elementary school-level fundraising restrictions are related to state law and district policy, but relevant policies and laws at the state and district level are not consistently implemented in schools. Across the board, the combination of policy at both the state and district level was significantly associated with a doubled likelihood of having a school policy, suggesting that district policies are helping to reinforce the state laws in this area, and that policy at both jurisdictions increases the likelihood of school-level restrictions. For overall fundraising restrictions, having a policy at only the district level was also associated with school-level policies; however, laws at the state level only (in the absence of district policies) were not associated with school-level policies. It is possible that districts are more effective at conveying information to the school-level than are states, or that enforcement provisions are stronger at the district level. These possibilities warrant further examination as to why school-level policies are more consistent with relevant district policies, but state laws are not.

Elementary schools located within districts and states where both had a strong policy/law were more than twice as likely to impose nutritional restrictions on fundraisers, to prohibit FMNVs in fundraising, and to prohibit soda in fundraising activities. In addition, specific provisions within the policies/laws were also associated with school-level restrictions, both for FMNVs and for soda. Policies banning candy and soda were positively associated with the presence of a school-level limit on FMNVs, and policy restrictions on soda were associated with school-level bans on the use of soda and/or soft drinks in fundraising activities. Nevertheless, further analysis indicated that elementary school-level policies were not necessarily consistent with district/state policies. Where district policy and state law required fundraising limitations, only 55.8% of schools reported having a school-level policy. The percentage of schools with a school-level policy was higher among schools in the West versus the South, and among those with a majority of Latino students versus a majority of White or Black students. The reasons for these variations are unclear but warrant further examination, as it will be important to better understand the barriers to school-level implementation of district policies and state laws. Some possibilities include concerns about lost revenue associated with implementing fundraising restrictions, regional variations in enforcement of policies, or that certain states and districts are more effective at conveying policy information to the school level [33].

Encouragingly, districts are continuing to develop and strengthen their wellness policies [28], [34] and research is accumulating to show that district policies and state laws are associated with school practices. For example, district policies are associated with reduced availability of junk foods and sugar-sweetened beverages in schools [35], [36]. Studies in individual states have shown significant improvements in the school food environment following development of laws and policies pertaining to foods in schools [22], [37], [38]. And, policies pertaining to competitive foods and beverages are associated with healthier dietary behaviors and weight outcomes among students [19].

Nevertheless, much more research is needed on the barriers to implementation of state laws and district policies pertaining to fundraising. With many school districts facing severe and ongoing economic challenges, schools and parent-teacher organizations are genuinely in need of strategies to raise funds for equipment and student activities, but for public health reasons it is important to raise money in ways that do not compromise students' health. Fortunately, however, there are other options for schools to raise funds that are both healthy and profitable. According to a report from the Center for Science in the Public Interest, walk-a-thons, book fairs, recycling fundraisers, auctions and car washes are all profitable fundraising options [39].

The health consequences of consuming sugary, high-calorie products are well documented. Given the national epidemic of childhood obesity and the importance of providing healthy school environments for young children, schools should consider using non-food-related fundraisers [39] or to ensure that any food-related fundraisers adhere to nutrition standards applied to items sold in other competitive food venues such as á la carte lines and vending machines [1]. As the USDA develops regulations and technical assistance materials related to wellness policies, as well as competitive food and beverage standards as required by the *Healthy, Hunger-Free Kids Act of 2010* [24], an opportunity exists to include provisions related to the nutritional content of food/beverage items sold through in-school fundraisers. In addition, more research is needed on the student-level impact of fundraising practices (i.e., actual student-level consumption habits and weight outcomes).

### Limitations

Relative to other data sources, these estimates are based on large numbers of schools over two school years, and the nationally-representative sample allows for inference to school practices across the country. However, there are also several potential limitations to this research. As with any survey that relies upon reported rather than observed data, it is possible that the estimates were affected by various reporting biases (e.g., desirability bias, response bias); however, the weights were adjusted to account for schools' propensity for non-response. The district fundraiser policies and state laws were assessed based on their inclusion in the sampled districts' congressionally-mandated wellness and related policies and codified state laws, respectively. In other words, we evaluated formal policies “on the books” rather than any informal guidelines, parent/student handbooks, or policies “in practice” that may also exist at the district and/or state levels. Thus, the estimates of district policy prevalence are conservative but given the relationships observed here, we expect that any additional “informal” policies would only further solidify the relationship between these policies and school-level restrictions. One of the dimensions for the district and state coding scheme addressed fundraising restrictions on “candy,” which we mapped to school-level FMNV restrictions; this is not a perfect match because although FMNVs include some types of candy, they do not include chocolates, which are commonly used in fundraisers. Thus, the policy dimension was broader than the school-level outcome, but nevertheless both were associated.

The school survey inquired about “school-wide policies,” but this does not guarantee that such policies would always have been enforced. In other words, respondents may have indicated that the school had a policy, but fundraising activities conducted by the parent-teacher organization or other groups might not have actually followed that requirement. Some respondents may have interpreted the item as regarding only policies that were developed at the school level, thus answering “no” where the school followed district policy but did not have a separate formal school policy, per se; however, some schools answered this item affirmatively then wrote in that they “follow district policy,” and our pre-testing of the survey with school principals did not reveal comprehension difficulty for this item. The goal of the school item was not to identify the source of the policy, but rather to assess whether the school had a policy or not. Nevertheless, some under-reporting of school-level policies (in districts that actually did have relevant policies) may have occurred; unfortunately we could not discern whether some of the schools in states/districts with policies that reported not having a school policy either had mis-reported their policy status or whether they truly did not know of or did not have a school policy. We also did not inquire about frequency or type of fundraising activity; in other words, our study addressed school policies, but not actual practices. There is much variability in practices, for example, in the frequency and type of fundraising activities; there is certainly an important difference between a school having a monthly bake sale in which high-calorie sugary items are offered directly to students versus a yearly school fundraiser that involves door-to-door sales of cookie dough or other products. Finally, we were unable to assess the magnitude of student exposure to energy-dense products—nor of the impact on weight outcomes due to student consumption of such products—through fundraising, but focused instead on school-wide fundraising restrictions. Nevertheless, the results do demonstrate the strong association between school policies with state law and district policy, and the continued need to promote school-level implementation of policies.

### Conclusions

In summary, many U.S. public elementary schools do not place nutritional restrictions on school fundraising activities. Few districts and states have strong policies about the nutritional quality of products used in fundraisers but, when they do, policies at both jurisdictions are associated with school-level restrictions. Revision of formal district policies and state laws to strengthen existing provisions written as recommendations rather than restrictions may be an effective strategy for impacting the elementary school environment, as would the development of new policies and laws where none exist. The USDA also has an opportunity through the forthcoming competitive food regulations to ensure that the standards apply broadly to all competitive foods sold in schools, including in-school fundraisers. Additional efforts are required to help schools follow district policies and state laws.
